# Frequency and characteristics of dysautonomic symptoms in multiple sclerosis: a cross-sectional double-center study with the validated Italian version of the Composite Autonomic Symptom Score-31

**DOI:** 10.1007/s10072-020-04620-1

**Published:** 2020-08-10

**Authors:** Matteo Foschi, Giulia Giannini, Elena Merli, Luca Mancinelli, Corrado Zenesini, Beatrice Viti, Pietro Guaraldi, Pietro Cortelli, Alessandra Lugaresi

**Affiliations:** 1 U.O.C. Neurologia - Ospedale S. Maria delle Croci, AUSL Romagna - ambito di Ravenna, Ravenna, Italy; 2 Dipartimento di Scienze Biomediche e Neuromotorie, Università di Bologna, Bologna, Italy; 3 Clinica Neurologica Rete Neurologica Metropolitana - IRCCS Istituto delle Scienze Neurologiche di Bologna, Ospedale Bellaria, Via Altura 3A, BO 40139 Bologna, Italy; 4 U.O.C. Neurologia - Ospedale Maurizio Bufalini, AUSL Romagna - ambito di Cesena, Cesena, Italy; 5 Unità di Epidemiologia e Statistica - IRCCS Istituto delle Scienze Neurologiche di Bologna, Ospedale Bellaria, Via Altura 3A, BO 40139 Bologna, Italy; 6 Divisione di Neurologia - Ospedale Infermi, AUSL Romagna - ambito di Rimini, Rimini, Italy; 7 UOSI Riabilitazione Sclerosi Multipla, IRCCS Istituto delle Scienze Neurologiche di Bologna, Ospedale Bellaria, Via Altura 3A, 40139 Bologna, BO Italy

**Keywords:** Multiple sclerosis, Dysautonomia, COMPASS-31, EDSS, Disease activity, Progression

## Abstract

**Background:**

Dysautonomic symptoms (DS) are frequent but often underrecognized in multiple sclerosis (MS) patients, despite the relevant impact on quality of life and physical performance.

**Objectives:**

To assess frequency and characteristics of DS in our MS population compared with healthy controls (HC). To investigate the relationship between DS and disease characteristics (MS subtype, disease duration, Expanded Disability Status Scale (EDSS), clinical and/or radiological activity, disability progression).

**Patients and methods:**

Cross-sectional study includes 324 MS patients (mean age 44.9 ± 10.7 years; 66% female) and 190 HC (mean age 40.60 ± 12.83 years; 63% female). DS were assessed using the Italian validated version of the Composite Autonomic Symptom Score-31 (COMPASS-31). Possible confounding factors were considered.

**Results:**

More than 94% of enrolled MS patients reported alterations in ≥ 2 domains of the COMPASS-31 scale (score > 0) and significantly higher COMPASS-31 total and single domain median scores compared with HC, independently from possible confounding factors (orthostatic intolerance: *p* = 0.001; vasomotor: *p* = 0.017; secretomotor: *p* = 0.040; gastrointestinal: *p* = 0.047; bladder: *p* < 0.001; pupillomotor: *p* < 0.001; COMPASS-31 total score: *p* < 0.001). COMPASS-31 total, secretomotor, gastrointestinal, and bladder domain scores showed weak to moderate correlation with disease duration (Rho = 0.19, *p* < 0.001; Rho = 0.18, *p* = 0.01; Rho = 0.25, *p* = 0.030; Rho = 0.28, *p* < 0.001, respectively). A moderate correlation between EDSS score, COMPASS-31 total, and bladder domain scores (Rho = 0.32, *p* < 0.001 and Rho = 0.48, *p* < 0.001, respectively) was observed. Progressive subtypes showed higher COMPASS-31 total (*p* = 0.025), gastrointestinal (*p* = 0.07), and bladder (*p* < 0.001) domain scores vs relapsing-remitting patients.

**Conclusions:**

Our findings confirm that MS-related DS are frequent and tend to increase paralleling disease duration and clinical worsening, reaching the highest clinical impact in progressive subtypes.

**Electronic supplementary material:**

The online version of this article (10.1007/s10072-020-04620-1) contains supplementary material, which is available to authorized users.

## Background

Multiple sclerosis (MS) is a complex immune-mediated and neurodegenerative disorder currently affecting about 2.3 million people worldwide with a mean age at onset of 30 years [1]. Since the earlier stages, demyelination and axonal loss can cause neurological symptoms including visual impairment due to optic neuritis (ON), motor and sensory disturbances, fatigue, bowel and bladder dysfunctions, and cognitive impairment [2]. Autonomic nervous system (ANS) function is frequently altered in MS patients not only as a consequence of spatial dissemination of demyelinating lesions but also in relation to changes due to concomitant medications or the development of central chronic deregulation throughout disease course [3]. The pathophysiology underlying dysautonomia in MS is probably related to the involvement of specific central nervous system (CNS) areas playing a direct or indirect role in autonomic function regulation [3, 4]. ANS abnormalities have been recently identified as predictors of disease activity, suggesting a possible role in regulating cellular and humoral immune function and in signaling the presence of inflammation to CNS [5]. The dysautonomic burden seems to increase paralleling lesion accumulation over time [6]. Notwithstanding the significant impact on patient’s quality of life and physical performance, the commonly used disability scale (Expanded Disability Status Scale (EDSS)) is mainly focused on motor and sensory symptoms. Hence, dysautonomic symptoms (DS), excluded sphincter disturbances, are often overlooked during routine consultations.

The use of self-reported questionnaires evaluating ANS functions has been demonstrated to improve the recognition of symptomatic dysautonomia [7, 8]. In particular, the Composite Autonomic Symptom Score-31 (COMPASS-31) is a self-reported questionnaire, created by the Autonomic Group of the Mayo Clinic, assessing autonomic disturbances [7]. It includes 31 items assessing 6 domains of autonomic nervous system dysfunction (orthostatic intolerance (OI), 4 items; vasomotor, 3 items; secretomotor, 4 items; gastrointestinal (GI), 12 items; bladder, 3 items; pupillomotor, 5 items). Scores from questions in each domain are added to obtain a raw domain score. The final domain score is generated by multiplying the raw score with a weight index. The total score is the sum of all domain scores and ranges from 0 (normal) to 100 (most severe dysautonomia) [7]. The reliability of COMPASS-31 as a self-assessment instrument for the detection of DS in MS patients has been already demonstrated by Drulović et al. [8]. The Italian version has been validated in 2015 by Pierangeli et al. by means of a standardized forward and expert panel back-translation procedure [9]. Studies based on the Italian version of COMPASS-31 to assess MS-related dysautonomia are currently lacking.

## Objectives

The primary aim of our study was to determine the frequency and characteristics of DS in our MS population in comparison with a population of healthy controls (HC) in order to assess the impact of MS in determining dysautonomia. The secondary aim was to investigate the relationship between DS and disease characteristics (MS subtype, disease duration, EDSS score, clinical and/or radiological activity, disability progression).

## Patients and methods

### Study design

The study design was cross-sectional. The Strengthening the Reporting of Observational Studies in Epidemiology (STROBE) guidelines were followed [10]. We consecutively enrolled patients referred to two MS centers (“U.O.S.I. Riabilitazione Sclerosi Multipla, IRCCS Istituto delle Scienze Neurologiche di Bologna” and “Divisione di Neurologia, Ospedale Infermi di Rimini”) from January 2017 to December 2018. Inclusion criteria were the following: (1) clinically definite MS (CDMS) according to the revised McDonald’s criteria [11] (patients with clinically isolated syndrome (CIS) were not included), (2) age between 18 and 65 years, and (3) ability to give verbal and written informed consent. Exclusion criteria included the following: (1) clinical relapse or corticosteroid (CCS) treatment in the last 30 days; (2) the presence of severe cardiological, endocrinological, GI, or psychiatric comorbidity; and (3) current pregnancy or parturition during the last year. Healthy controls (HC) were recruited mainly among patients’ significant others or caregivers, excluding consanguineous relatives and subjects with a family history positive for MS. The same age, comorbidity, and pregnancy exclusion criteria were applied. Every subject enrolled was encouraged to fill out the COMPASS-31 questionnaire (briefly described in the background section) independently, to avoid investigators’ biases. Question rephrasing and further explanations were provided upon request.

The following clinical data were collected in a standardized fashion by 5 clinicians: disease duration, MS subtype (relapsing-remitting (RR), primary progressive (PP), or secondary progressive (SP)), presence of clinical or radiological activity (defined by evidence of relapses or new/enlarging/gadolinium (Gd) enhancing lesions in a brain or spine magnetic resonance imaging (MRI) scan), and presence of disease progression (6-month confirmed increase of ≥ 1.5 for EDSS 0, ≥ 1 for EDSS 1–5.0, ≥ 0.5 for EDSS > 5.0 in the EDSS score) during the last year. In addition, possible major confounding factors, in relation to MS and DS, were considered, such as symptomatic medications possibly interfering with ANS function (e.g., anticholinergics, antidepressant, antihypertensive, beta-blockers, diuretics, antiarrhythmics, opioids). Patients without major confounding factors were included in the P subgroup, whereas the remaining patients constituted the P* subgroup. Further analyses were adjusted considering current cigarette smoking habit (given the parasympathomimetic effect of nicotine) [12], sex, and age as potentially influencing the questionnaire results both in HC and P-P* subgroups. The impact of disease-modifying therapies (DMTs) on ANS function was tested comparing specific domain scores of treated and untreated patients, in relation to well-known side effects [13]. These included (1) flushing and GI disturbances for dimethyl fumarate (DMF) (vasomotor and GI domains), (2) flu-like symptoms for interferon beta (IFNB) (sudomotor, vasomotor, and GI domains), and (3) enhanced cardiovagal tone for fingolimod (FTY) (OI domain).

### Statistical analysis

Normality of continuous variable distribution was checked using the Skewness-Kurtosis test. Continuous variables are presented as mean ± standard deviation (SD) or median and interquartile range (IQR) based on variable distribution. Categorical variables are presented as absolute (*n*) and relative frequency (%). The Mann-Whitney *U* test, Kruskal–Wallis test, or Student’s *t* test were used to compare continuous variables. The Bonferroni post hoc correction was used for multiple comparisons between subgroups. Categorical variables were compared by Chi-square test. Confounding factors were controlled using a two-step approach: (1) applying a restriction to the MS group for major confounders (symptomatic medications possibly interfering with ANS function) and (2) using multivariable Poisson regression models (one for each domain and one for the total score) with COMPASS-31 scores as dependent variables and group with confounding factor (P) vs. HC as independent variable (HC was the reference category), adjusted for other possible confounders (age, sex, cigarette smoking habit). Results are presented as incidence rate ratio (IRR) and relative 95% confidence interval (95% CI). The Spearman’s Rho coefficient was used to evaluate the correlation between continuous variables. Statistical analysis was performed using the statistical package Stata SE, 14.2.

## Results

**Fig. 1:**
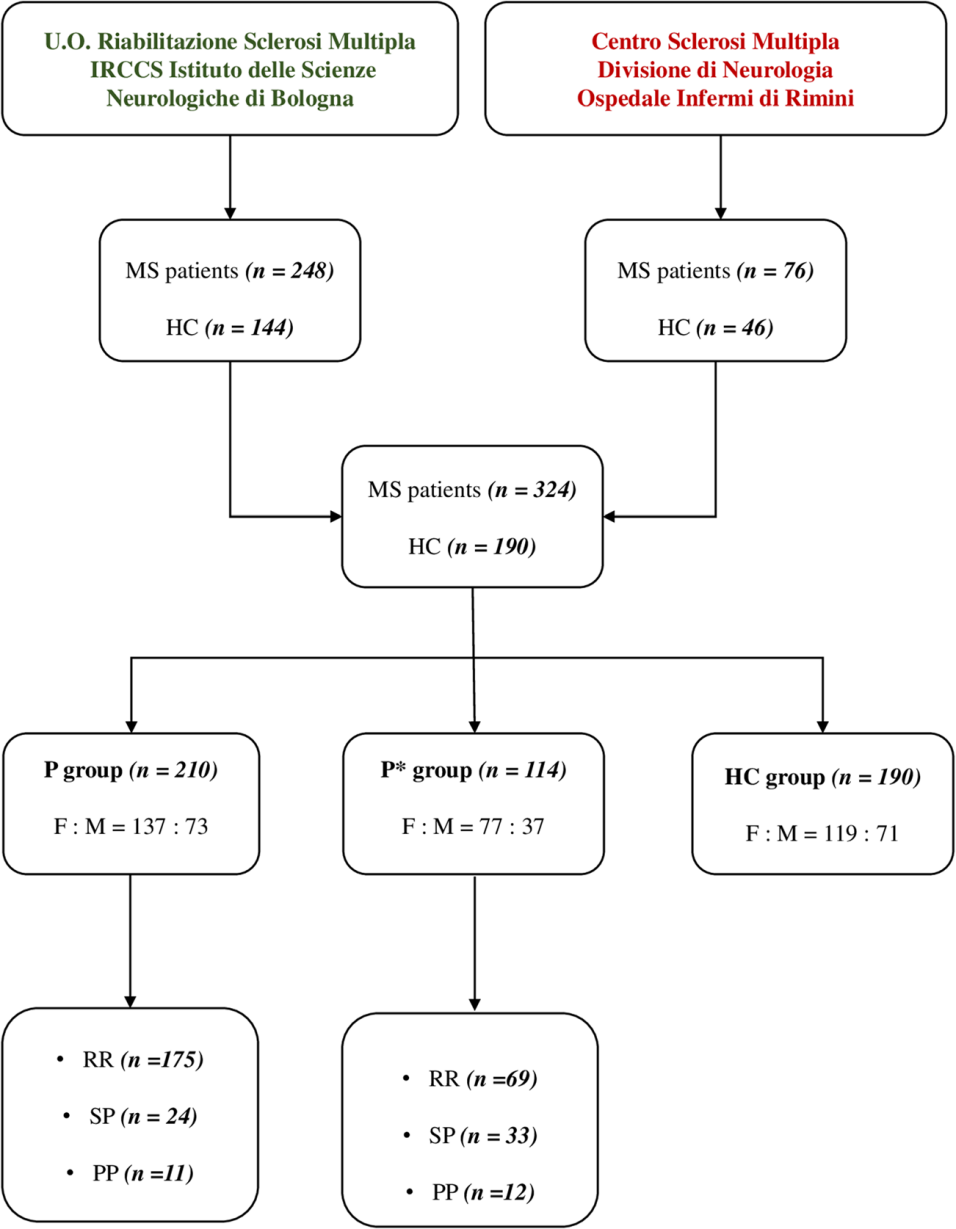
Study flow chart. MS, multiple sclerosis; HC, healthy controls; RR, relapsing-remitting; SP, secondary progressive; PP, primary progressive

We enrolled 324 MS patients and 190 HC. Out of the 324 patients, 210 (65.6%) were included in the P group, and the remaining 114 (34.4%) formed the P* group. The study flow chart is illustrated in Fig. 1.

### Demographic and clinical characteristics

MS patients included 214 females (66%) and 110 males (34%) with a mean age of 44.9 ± 10.7 years. Two hundred forty-four patients (75.7%) had relapsing-remitting MS (RRMS), 57 (17.5%) secondary progressive MS (SPMS), and 23 (7.1%) primary progressive MS (PPMS). Median disease duration was 12 years (IQR 6–19), and median EDSS score was 3.2 (IQR 0–7.5). One hundred twenty-one patients (37.4%) had a positive history for optic neuritis (ON). Sixty patients (29%) were active smokers. Demographic and clinical characteristics of P and P* subgroups and HC are reported in Table 1.

**Table 1:** Demographic and clinical characteristics of multiple sclerosis patients and healthy controls

Demographic characteristics	P (*n* = 210)	P* (*n* = 114)	HC (*n* = 190)
Sex, female—*N* (%)	137 (65)	77 (68)	119 (63)
Age, years*—*mean (SD)	43.0 (10.5)	48.4 (10.4)	40.6 (12.8)
Cigarette smoking—*N* (%)	60 (29)	44 (39)	39 (21)
Clinical characteristics	P (*n* = 210)	P* (*n* = 114)	*p* value
Disease factors			
Disease duration—median (IQR)	11 (6–19)	14 (7–22)	0.019
1-year activity—*N* (%)	94 (45)	47 (41)	0.540
1-year progression—*N* (%)	32 (15)	33 (29)	0.003
EDSS—median (IQR)	2 (1–4)	4 (2.5–6)	< 0.001
MS course—*N* (%)			
Relapsing-remitting	175 (83)	69 (60)	< 0.001
Secondary progressive	24 (12)	33 (29)	
Primary progressive	11 (5)	12 (11)	
Disease history—*N* (%)			
History of optic neuritis	85 (40)	36 (32)	< 0.001
History of myelitis	17 (8)	9 (8)	0.941
Disease-modifying therapy—*N* (%)			
Interferon beta	56 (27)	18 (16)	0.026
Glatiramer acetate	29 (14)	19 (17)	0.716
Dimethyl fumarate	13 (6)	10 (9)	0.388
Teriflunomide	9 (4)	6 (5)	0.498
Fingolimod	11 (5)	5 (4)	0.992
Natalizumab	16 (8)	6 (5)	0.420
Alemtuzumab	2 (1)	1 (1)	0.946
Ocrelizumab	3 (1)	0 (0)	0.200
Rituximab	0 (0)	1 (1)	0.174
Azathioprine	5 (2)	9 (8)	0.020
None	61 (29)	36 (32)	0.262

*HC* healthy controls, *n* number of subjects, *IQR* interquartile range, *SD* standard deviation. *p* value = Mann-Whitney *U* test for continuous variables, Chi-square test for categorical variables for comparisons between groups with (P*) and without (P) confounding factors

### Dysautonomic symptoms

We observed that 318 out of 324 MS patients (98.1%) and 188 out of 190 HC (99%) reported alterations in ≥ 1 domains of the COMPASS-31 scale (score > 0). An alteration in ≥ 2 domains was reported by 94.8% of MS patients (307 out of 324) and 85.3% of HC (162 out of 190 HC). Patients with MS showed significantly higher median COMPASS-31 total score and single domain scores in comparison with HC, either considering the whole MS population (Table 2A) or only patients without confounding factors (P group, Table 2B). The multivariable Poisson regression models confirmed the higher scores of the P group vs. HC group even after adjusting for age, sex, and cigarette smoking habit (Table 3). The highest relative incidence rate ratios were observed for bladder domain scores (IRR = 5.2, 95% CI = 4.0–6.7) and vasomotor domain scores (IRR = 3.4, 95% CI = 3.2–5.2).

**Table 2:** Frequency of dysautonomic symptoms and COMPASS-31 total and single domain scores

A	MS patients—324 patients		HC—190 subjects		*p* value
COMPASS-31	*N* (%)	Median (IQR)	*N* (%)	Median (IQR)	
Orthostatic	175 (54.2)	8 (0–16)	75 (39.5)	0 (0–12)	< 0.001
Vasomotor	70 (21.7)	0 (0–0)	11 (5.8)	0 (0–0)	< 0.001
Secretomotor	149 (46.1)	0 (0–6.4)	55 (29.0)	0 (0–2.1)	< 0.001
Gastrointestinal	294 (90.7)	5.4 (2.7–8.9)	185 (97.4)	4.5 (2.7–6.3)	< 0.001
Bladder	211 (65.3)	1.1 (0–3.3)	44 (23.2)	0 (0–0)	< 0.001
Pupillomotor	259 (80.2)	1.7 (1–2.7)	146 (76.8)	1 (0.7–1.7)	< 0.001
Total score	–	20.6 (8.1–34)	–	10.2 (4.7–19.7)	< 0.001
B	P—210 patients		HC—190 subjects		*p* value
COMPASS-31	*N* (%)	Median (IQR)	*N* (%)	Median (IQR)	
Orthostatic	108 (51.4)	8 (0–16)	75 (39.5)	0 (0–12)	0.001
Vasomotor	41 (19.5)	0 (0–0)	11 (5.8)	0 (0–0)	0.017
Secretomotor	84 (40.0)	0 (0–4.3)	55 (29.0)	0 (0–2.1)	0.040
Gastrointestinal	190 (90.5)	5 (1.8–8)	185 (97.4)	4.5 (2.7–6.3)	0.047
Bladder	119 (56.5)	1.1 (0–3.3)	44 (23.2)	0 (0–0)	< 0.001
Pupillomotor	169 (80.5)	1.7 (1–2.3)	146 (76.8)	1 (0.7–1.7)	< 0.001
Total score	–	16.6 (7–32)	–	10.2 (4.7–19.7)	< 0.001

*N* (%) number of patients reporting a score > 0 (percentage of the total), *IQR* interquartile range. Comparison between (A) multiple sclerosis (MS) patients and healthy controls (HC) and (B) MS patients without confounding factors (P group) and HC. *p* value = Mann-Whitney *U* test for comparison between the group without confounding factors (P) and HC; the null hypothesis is no difference between groups

**Table 3:** COMPASS-31 total and domain score comparison between MS patients without confounding factors (P group) and healthy controls (HC) adjusted for age, sex, and cigarette smoking

COMPASS-31	IRR (95% CI)	*p* value
Orthostatic	1.7 (1.6–1.8)	< 0.001
Vasomotor	3.4 (2.2–5.2)	< 0.001
Secretomotor	1.3 (1.2–1.7)	< 0.001
Gastrointestinal	1.2 (1.1–1.3)	< 0.001
Bladder	5.2 (4.0–6.7)	< 0.001
Pupillomotor	1.3 (1.1–1.5)	0.002
Total score	1.5 (1.4–1.6)	< 0.001

*IRR* incidence rate ratio, *95% CI* 95% confidence interval. Multivariable Poisson regression models (one for each domain and one for the total score) with COMPASS-31 scores as dependent variable and group without confounding factors (P) vs. HC as independent variable, adjusted for age, sex, and cigarette smoking. *p* value = the null hypothesis is IRR = 1, no difference between groups P and HC

Considering the whole MS group, comparison between sexes showed higher median scores in women for all domains except bladder (*p* = 0.951) and vasomotor (*p* = 0.278), with a significant difference for OI (12 IQR 0–20 F vs. 0 IQR 0–16 M, *p* = 0.008), secretomotor (1.1 IQR 0–6.4 F vs. 0 IQR 0–4.3 M, *p* = 0.037), GI (6.3 IQR 3.6–9 F vs. 4.5 IQR 1.8–7 M, *p* = 0.002), pupillomotor (1.7 IQR 1–2.7 F vs. 1.3 IQR 0.7–2 M, *p* = 0.001) domains, and for COMPASS-31 total score (24 IQR 10.5–37 F vs. 13.8 IQR 6.5–30 M, *p* = 0.001).

### Effects of disease factors

Regarding disease factors and considering the whole MS population, Spearman’s coefficient indicated a weak to moderate correlation between disease duration, COMPASS-31 total score (Rho = 0.19, *p* < 0.001), secretomotor (Rho = 0.18, *p* = 0.01), GI (Rho = 0.25, *p* = 0.030), and bladder domains (Rho = 0.28, *p* < 0.001) (Table 4). A moderate correlation between EDSS score, COMPASS-31 total score (Rho = 0.32, *p* < 0.001), and bladder domain (Rho = 0.48, *p* < 0.001) was observed. Most of these correlations were confirmed (although with lower Rho values) even considering only patients without confounding factors. We observed a significant trend to report higher bladder, GI, and COMPASS-31 total scores in progressive disease subtypes compared with RR-MS (*p* < 0.05) (Table 5). Demographic and clinical characteristics of progressive and RR subgroups are shown in Supplementary Table 1. Adjusting for age, sex, disease duration, and EDSS score, the significant difference between COMPASS-31 total, bladder, and GI domain scores of progressive and RR disease subtypes was not confirmed (Supplementary Table 2). Significantly higher secretomotor (*p* = 0.034), bladder (*p* < 0.001), and COMPASS-31 total scores (*p* = 0.045) were observed in patients with a recent history of MS progression (Supplementary Table 3). No significant intergroup differences were observed in relation to the presence, in the last year, of recent clinical/radiological evidence of disease activity (Supplementary Table 4). Patients with a positive history for ON showed higher scores in the pupillomotor domain compared with patients negative for ON and HC (*p* < 0.001). The difference in the pupillomotor domain between ON-negative MS patients and HC remained significant (*p* < 0.001) (Supplementary Table 5A). The comparison between COMPASS-31 total and single domain scores between patients with or without a positive history for myelitis did not show significant differences (Supplementary Table 5B). Regarding patients treated with DMTs possibly impacting autonomic function (IFNB, DMF, fingolimod) compared with untreated patients, we found no significant differences (Supplementary Table 6).

**Table 4:** Spearman’s correlation between COMPASS-31 total and domain scores, disease duration, and EDSS in the total sample of 324 MS patients

COMPASS-31	Disease duration Rho (*p* value)	EDSS Rho (*p* value)
Orthostatic	0.062 (0.264)	0.193 (< 0.001)
Vasomotor	0.059 (0.287)	0.147 (0.008)
Secretomotor	0.177 (0.001)	0.228 (< 0.001)
Gastrointestinal	0.250 (0.030)	0.204 (0.030)
Bladder	0.283 (< 0.001)	0.477 (< 0.001)
Pupillomotor	0.054 (0.336)	0.057 (0.309)
Total score	0.190 (< 0.001)	0.323 (< 0.001)

*MS* multiple sclerosis, *EDSS* Expanded Disability Status Scale. *p* value: the null hypothesis was the Spearman’s Rho = 0, absence of correlation. COMPASS-31 total, secretomotor, gastrointestinal, and bladder domain scores showed a weak to moderate correlation with disease duration. COMPASS-31 total and bladder domain scores showed a moderate correlation with EDSS

**Table 5:** COMPASS-31 total and domain score comparison between different clinical courses of MS in the total sample of 324 patients

COMPASS-31	RR - *N* = 244 Median (IQR)	SP - *N* = 57 Median (IQR)	PP - *N* = 23 Median (IQR)	*p* value
Orthostatic	8 (0–16)	12 (0–20)	12 (0–20)	0.488
Vasomotor	0 (0–0)	0 (0–1.7)	0 (0–0)	0.240
Secretomotor	0 (0–4.3)	2.1 (0–6.4)	0 (0–6.4)	0.170
Gastrointestinal	5.4 (2.7–8)	7.1 (4.5–9.8)	5.4 (3.6–7.1)	0.070
Bladder	1.1 (0–2.2)	3.3 (1.1–5.6)	2.2 (1.1–4.5)	< 0.001
Pupillomotor	1.7 (1–2.7)	1.3 (0.3–2)	1 (0–2.3)	0.187
Total score	18.9 (7.3–32)	22.6 (12–42.3)	30 (7.4–39.2)	0.025

*PP* primary progressive, *SP* secondary progressive, *RR* relapsing-remitting, *IQR* interquartile range. *p* value = Kruskal–Wallis test for comparison between RR, SP, and PP groups; the null hypothesis is no difference among the three groups

## Discussion

Our cross-sectional study was based on the administration of the Italian-validated version of COMPASS-31. We evaluated frequency and characteristics of DS in a large group of MS patients (*n* = 324) attending two Italian MS centers. Our MS sample included patients with late CDMS, long disease duration (14 ± 9.4 years), and a mean age of 44.9 ± 10.7 years. CIS patients were excluded from the study. MS patients showed increased COMPASS-31 total and single domain scores when compared with HC (*p* < 0.001, Table 2A). Significantly higher scores were observed also taking into consideration only MS patients without symptomatic medications possibly affecting ANS function such as anticholinergic drugs (Table 2B), suggesting a causal relationship between MS and DS. The most relevant differences with HC were found for OI, bladder, and pupillomotor domains (*p* < 0.001). These results are reasonably driven by an injury to specific brain or spinal cord ANS pathways (e.g., increased bladder and GI scores) or by a direct damage to the pupillary system due to ON (increased pupillomotor score), although changes in autonomic function independent from MS cannot be excluded. Indeed, although in our MS population patients treated with DMTs possibly associated with dysautonomic side effects (IFNB, DMF, FTY) [13] showed similar median scores in comparison with untreated patients (Supplementary Table 5), subtle drug-dependent impairment of autonomic function cannot be excluded. In this regard, the small size of DMT subgroups, along with dissimilarities in demographic and MS course characteristics, might hamper the detection of subtle differences in DS scores between treated and untreated patients. Finally, a constant reorganization of ANS networks throughout MS course could lead to central chronic deregulation contributing to the development of dysautonomia [14], especially in a population with moderate disease severity (median EDSS: 3.2 IQR 0–7.5), older age, and long disease, as in our sample.

Concerning the frequency of DS, we have shown that, using the COMPASS-31 questionnaire in adults, subtle ANS impairment in at least 1 domain is present in the majority (> 98%) of subjects, independently from the diagnosis of MS. On the contrary, the presence of a score > 0 in ≥ 2 domains was more common in MS patients compared with HC (94.8% vs. 85.3%, respectively). The percentage of patients reporting DS in our MS population is in line with the high prevalence (97%) reported by Vieira et al. among 133 RR-MS patients using the Portuguese version of the COMPASS-31 [15]. The most frequently involved domain in our MS population was the GI (90.7%). This observation supports data from other studies reporting slower gastric emptying, bowel dysmotility, constipation, or fecal incontinence as common findings in MS [16–18]. Noticeably, a high proportion of HC showed a GI subscale score > 0 (97.4%). This finding might derive from distress and over-sensitization concerning health problems among partners and caregivers of MS patients included in the HC group. Additionally, it can be hypothesized that the GI domain is too sensitive and might elicit positive answers even in the presence of anxiety or other confounders, in the absence of clear autonomic dysfunction. Secretomotor symptoms such as altered sweating, dry eyes, or dry mouth have been reported to affect about 46.1% of the MS population [19]. We observed a slightly lower prevalence (41%) in the P group, which excluded patients taking anticholinergic medications. Prevalence of OI in the P group (51.4%) was also in line with results of prior studies including ANS cardiovascular function objective assessment (e.g., heart rate variability with deep breathing or head-up tilt test) [20]. As observed elsewhere [15], we confirmed that bladder disturbances (urinary urgency or retention) occur frequently during the disease course (65.3%). Interestingly, we observed a higher pupillomotor domain score compared with HC even in patients without a prior history of ON. Formal ophthalmological evaluations were unfortunately not performed to exclude nonneurologic visual defects. In particular, most of our MS patients reported sensitivity to bright light or accommodation defects (80.2%) even in the absence of prior ON (present in about 40% of cases). This finding is suggestive of a specific autonomic dysfunction and not of an afferent defect and is in line with the high prevalence of parasympathetic efferent defects reported by De Seze et al. independently from concomitant visual evoked potential alterations [21]. The underlying pathogenic mechanism remains poorly characterized but seems to be related to axonal loss along pupillomotor fibers consistent with the evidence of a correlation between efferent pathway shift and spinal cord atrophy [21]. Furthermore, Crnošija et al. observed substantial differences in characteristics of pupillomotor disturbances comparing 20 matched patients with MS and neuromyelitis optica spectrum disorder (NMOSD) as assessed by COMPASS-31 [22]. While pupillomotor abnormalities in MS patients may reflect parasympathetic pupillary dysfunction with a relative increase of sympathetic dilatator tone [21, 22], the more severe subtype of ON in NMOSD could determine either a permanent visual loss with consequently altered pupillary responses or an extensive involvement of the pupillary response system [22]. We found no significant difference in COMPASS-31 total and single domain scores between patients with or without a positive history for myelitis. This observation could be explained considering the potential role of concomitant factors (e.g., age-related changes, brain atrophy, accrual of demyelinating lesions in different CNS areas, especially in the brainstem) occurring during the disease course and increasing the dysautonomic burden also in patients without spinal cord involvement.

Comparison between sexes showed that symptomatic dysautonomia was more severe in female patients in comparison with male, except for bladder dysfunction. This difference was less evident in HC, although higher mean scores were also reported by females, as reported by prior studies administering the same questionnaire in the healthy population [23]. This observation suggests that MS is likelier to cause DS in the context of subclinical or paucisymptomatic dysautonomia, especially in domains which are well known to be more frequently altered in female subjects (e.g., GI, secretomotor, OI) [23]. Moreover, our results highlight the importance of investigating the presence of autonomic dysfunction before prescribing symptomatic medications or DMTs possibly interfering with ANS function, especially in female MS patients. In these cases, the use of COMPASS-31 could help to identify patients with more pronounced baseline dysautonomia to be monitored more strictly during treatment [13].

Relative to the effects of disease factors, disease duration showed a weak to moderate correlation with COMPASS-31 total, secretomotor, GI, and bladder scores (Rho = 0.19, *p* < 0.001; Rho = 0.18, *p* = 0.01; Rho = 0.25, *p* = 0.03; Rho = 0.28, *p* < 0.001, respectively). A moderate correlation between EDSS score, COMPASS-31 total score (Rho = 0.32, *p* < 0.001), and bladder domain score (Rho = 0.48, *p* < 0.001) was also observed. These data suggest that the probability of developing symptomatic dysautonomia in MS increases with disease duration and disability accrual over time. Prior studies investigating the relationship between ANS involvement and disease-related parameters gave conflicting results. The prospective controlled study by Gunal et al. demonstrated a strong correlation between cardiovascular and skin sympathetic response abnormalities with disease duration and EDSS score in 22 RR-MS patients [24]. On the contrary, a larger study by Cortez et al. including 100 MS patients (84% RR, 12% SP, 4% PP) failed to show a significant correlation between COMPASS-31 total score, EDSS (Rho = 0; *p* = 0.97), and disease duration (Rho = 0.02; *p* = 0.84) [25]. Conflicting results might be explained by the small size of previous studies and other methodological issues. We also observed significantly higher COMPASS-31 total scores in SP and PP-MS patients, compared with RR-MS (*p* < 0.001). This finding was not confirmed by the multivariable Poisson regression models adjusting for factors possibly contributing to dysautonomia (F/M ratio, age, disease duration, and EDSS—Supplementary Table 2). Therefore, we can conclude that rather than the type of disease course, it is the difference in the demographic and clinical characteristics which plays a key role in determining the degree of ANS impairment. The observation of significantly higher COMPASS-31 total scores reported by patients with progressive subtypes or a recent history of MS progression (*p* < 0.001) suggests that the dysautonomic burden is more pronounced in advanced and progressive disease phases, possibly in relation to lesion accumulation in strategic CNS areas over time and concomitant widespread neurodegeneration. Consistent with our findings, the cross-sectional study by Adamec et al. disclosed a higher dysautonomic burden in progressive MS subtypes, using the Composite Autonomic Scoring Scale (CASS) and heart rate variability to compare 40 RR and 40 progressive MS patients [26]. In our study, with a mean disease duration longer than 10 years, patients with a history of disease activity in the previous 12 months did not show significantly higher COMPASS-31 total and single subscales mean scores in comparison with patients without recent clinical and/or radiological evidence of disease activity. The accumulation of significant ANS damage could have been present since the earliest disease stages. The cross-sectional design of our study does not allow inferences on the role of recent disease activity on the variation of ANS disturbances as assessed by the COMPASS-31. Prospective studies, such as the observational study by Krbot Skorić et al., showed that a higher COMPASS-31 score (> 7.32) was related with a lower probability of being relapse free (*p* = 0.013) [27]. To be noted that the study included only patients with CIS with a short mean disease duration (2.9 years) [27] not comparable to our population.

Limitations of our study include the cross-sectional design and the lack of formal ANS function assessment through objective gold standard tests. Moreover, sexual dysfunction is common in MS patients but not investigated in the COMPASS-31 (nor captured in the EDSS). Anyway, the detection of sphincter disturbances might serve as a proxy indicator and lead to direct questions to patients, to avoid overlooking a hidden, but relevant to quality of life, symptom.

## Conclusions

Our findings confirm that symptoms of autonomic dysfunction are frequent in MS and highlight the importance of direct assessment of ANS function. The Italian version of COMPASS-31 is a practical and easily self-administrable questionnaire able to assess the presence of DS in MS patients in all disease phases and courses. Its use may improve detection and management of dysautonomia, easily overlooked by routine neurological evaluation. Frequency and severity of DS increase paralleling disease duration and disability accumulation over time, especially for cardiovascular, GI, and bladder dysfunction. The highest clinical impact of autonomic impairment is observed in the more advanced stages and progressive MS subtypes, but we believe that the administration of COMPASS-31 might be particularly useful to detect and address subtle dysautonomia, expressing more a subtle impairment rather than irreversible autonomic pathway changes, especially in the early relapsing phase of MS. A prospective study investigating ANS function with the COMPASS-31 followed by formal autonomic testing is needed to strengthen the meaningfulness of our results and to confirm our hypotheses. To clarify whether ANS dysfunction in MS results from a direct damage to specific autonomic pathways, future investigations should incorporate MRI studies quantifying brain/spine atrophy, lesion load, and site.

## Electronic supplementary material

ESM 1 (DOCX 26 kb)

## Data Availability

Pseudonymised participant data and tables not included in the article will be made available upon request to the corresponding author.
