# Supplementary material for: Frequency and characteristics of dysautonomic symptoms in multiple sclerosis: a cross-sectional double-center study with the validated Italian version of the Composite Autonomic Symptom Score-31
Source: Neurol Sci. 2020 Aug 10;42(4):1395–403. doi: 10.1007/s10072-020-04620-1 (PMC7955976; doi:10.1007/s10072-020-04620-1)
Supplement: Supplementary file 1 — (DOCX 26 kb) [file 10072_2020_4620_MOESM1_ESM.docx]

**SUPPLEMENTARY TABLES**

**Supplementary Table 1. Demographic and clinical characteristics of patients with different clinical courses of MS**

P-value = Kruskal-Wallis test for comparison between groups RR, SP and PP, the null hypothesis is no difference between groups

|  | **RR**  **N = 244** | **SP**  **N = 57** | **PP**  **N = 23** | **P-value** |
| --- | --- | --- | --- | --- |
| **Female**  **N (%)** | 171 (70.1) | 35 (61.4) | 8 (36.4) | 0.002 *^1^ |
| **Age – yr**  **Median (IQR)** | 42 (35 – 50) | 54 (48 – 58) | 54 (52 – 59) | < 0.001*^1^ |
| **Disease duration**  **Median (IQR)** | 10 (6 – 18) | 24 (15 – 29) | 9 (5 – 18) | < 0.001*^2^ |
| **EDSS**  **Median (IQR)** | 2 (1 – 3.5) | 6 (5 – 6.5) | 5 (4 – 6.5) | < 0.001*^1^ |

RR = relapsing-remitting; SP = secondary progressive; PP = primary progressive; EDSS = Expanded Disability Status Scale

*^1^ Bonferroni post-hoc showed a significance difference between RR vs. SP and RR vs. PP

*^2^ Bonferroni post-hoc showed a significance difference between SP vs. RR and SP vs. PP

**Supplementary Table 2. Compass-31 total and domain scores comparison between different clinical courses of MS in the total sample of 324 patients adjusted for sex, age, disease duration and EDSS.**

Multivariable Poisson regression models (one for each domain and one for the total score) with COMPASS-31 scores as dependent variable and MS course as independent variable (RR as reference group), adjusted for sex, age, disease duration and EDSS. P-value = the null hypothesis is IRR = 1, absence of difference between groups

| **SP vs RR** | | | **PP vs RR** | |
| --- | --- | --- | --- | --- |
|  | **IRR (95% CI)** | **P-value** | **IRR (95% CI)** | **P-value** |
| **Orthostatic** | 0.9 (0.8 – 1.0) | 0.127 | 1.1 (1.0 – 1.3) | 0.118 |
| **Vasomotor** | 0.9 (0.6 – 1.4) | 0.595 | 0.9 (0.8 – 1.1) | 0.102 |
| **Secretomotor** | 0.8 (0.7 – 1.0) | 0.114 | 0.9 (0.7 – 1.2) | 0.388 |
| **Gastrointestinal** | 1.0 (0.8 – 1.1) | 0.601 | 1.0 (0.8 – 1.2) | 0.657 |
| **Bladder** | 0.9 (0.7 – 1.1) | 0.459 | 1.0 (0.7 – 1.3) | 0.867 |
| **Pupillomotor** | 0.7 (0.5 – 1.1) | 0.137 | 0.9 (0.6 – 1.3) | 0.563 |
| **Total score** | 0.9 (0.8 – 1.0) | 0.113 | 1.0 (0.9 – 1.1) | 0.958 |

RR = relapsing-remitting; SP = secondary progressive; PP = primary progressive; EDSS = Expanded Disability Status Scale; IRR = Incidence rate ratio, 95% CI = 95% Confidence Interval

**Supplementary Table 3. COMPASS-31 total and domain scores comparison between patients without (A) and with (B) recent (12-month) evidence of disease worsening in the total sample of 324 patients**

P-value = Mann-Whitney U-test for comparison between groups of patients without (A) and with (B) recent (12-month) evidence of disease progression, the null hypothesis is no difference between groups.

| **COMPASS-31** | **A - N = 258**  **Median (IQR)** | **B - N = 66**  **Median (IQR)** | **P-value** |
| --- | --- | --- | --- |
| **Orthostatic** | 8 (0 – 16) | 8 (0 – 20) | 0.628 |
| **Vasomotor** | 0 (0 – 0) | 0 (0 – 1.7) | 0.284 |
| **Secretomotor** | 0 (0 – 4.3) | 4.3 (0 – 6.4) | 0.034 |
| **Gastrointestinal** | 5.4 (2.7 – 8.9) | 6.3 (3.6 – 8) | 0.431 |
| **Bladder** | 1.1 (0 – 3.3) | 3.3 (1.1 – 4.4) | <0.001 |
| **Pupillomotor** | 1.7 (1 – 2.7) | 1.7 (1 – 2.3) | 0.894 |
| **Total score** | 18.8 (7.4 – 33) | 26.4 (11 – 38.5) | 0.045 |

IQR = interquartile range

**Supplementary Table 4. Compass-31 total and domain scores comparison between patients without (C) and with (D) recent (12-month) clinical/radiological evidence of disease activity in the total sample of 324 patients**

P-value = Mann-Whitney U-test for comparison between groups of patients without (C) and with (D) recent (12-month) clinical/radiological evidence of disease activity, the null hypothesis is no difference between groups

| **COMPASS-31** | **C - n =183**  **Median (IQR)** | **D - n = 141**  **Median (IQR)** | **P-value** |
| --- | --- | --- | --- |
| **Orthostatic** | 12 (0 - 20) | 8 (0 – 16) | 0.693 |
| **Vasomotor** | 0 (0 – 0) | 0 (0 – 0) | 0.484 |
| **Secretomotor** | 0 (0 – 6.4) | 0 (0 – 4.3) | 0.527 |
| **Gastrointestinal** | 5.4 (2.7 – 8.9) | 5.4 (2.7 – 8) | 0.878 |
| **Bladder** | 2.2 (0 – 3.3) | 1.1 (0 – 3.3) | 0.103 |
| **Pupillomotor** | 1.7 (1 – 2.3) | 1.7 (1 – 2.7) | 0.744 |
| **Total score** | 21.8 (8 – 35.2) | 19.6 (8.1 – 32.8) | 0.528 |

IQR = interquartile range

**Supplementary Table 5.**

**A): Pupillomotor score comparison between patients with (ON+) and without (ON-) prior history of optic neuritis in the total sample of 324 patients.**

P-value = Kruskal-Wallis test for comparison between groups ON+, ON- and HC, the null hypothesis is no difference between groups

| **A** | **ON + (N = 121)**  **Median (IQR)** | **ON - (N = 203)**  **Median (IQR)** | **HC – (N = 190) Median (IQR)** | **P-value** |
| --- | --- | --- | --- | --- |
| **Pupillomotor** | 2 (1.3 – 3) | 1.3 (0.3 – 2.3) | 1 (0.6 – 1.7) | < 0.001* |

*Bonferroni post-hoc showed a significance difference between ON+ vs. ON – and ON + vs. HC and ON - vs. HC

IQR = interquartile range, HC = healthy controls

**B): COMPASS-31 total and domain scores comparison between patients with (M+) and without (M-) history of myelitis in the total sample of 324 patients.**

P-value = Mann-Whitney U-test for comparison between patients without (M-) and with (M+) history of myelitis, the null hypothesis is no difference between groups.

| **B** | **M+ (N = 235)**  **Median (IQR)** | **M– (N = 89)**  **Median (IQR)** | **P-value** |
| --- | --- | --- | --- |
| **Orthostatic** | 8 (0 – 16) | 12 (0 – 20) | 0.266 |
| **Vasomotor** | 0 (0 – 0) | 0 (0 – 0) | 0.588 |
| **Secretomotor** | 0 (0 – 6.4) | 0 (0 – 4.3) | 0.324 |
| **Gastrointestinal** | 5.4 (2.7 – 8.9) | 6.3 (2.7 – 8.9) | 0.434 |
| **Bladder** | 1.1 (0 – 3.3) | 2.2 (0 – 3.3) | 0.105 |
| **Pupillomotor** | 1.7 (0.7 – 2.7) | 1.7 (1 – 2.3) | 0.929 |
| **Total score** | 18.1 (7.9 – 33.6) | 24 (8.7 – 35.3) | 0.350 |

IQR = interquartile range

**Supplementary Table 6. Comparison between domain scores of patients treated with disease-modifying therapies possibly affecting autonomic function and untreated patients.**

**A): Interferon-beta (IFNB); B): Dimethyl fumarate (DMF); C): Fingolimod (FTY).**

P-value = Mann-Whitney U-test for comparison between groups, the null hypothesis is no difference between groups

| **A** | **IFNB**  **(N = 74)**  **Median (IQR)** | **Untreated**  **(N = 97)**  **Median (IQR)** | **P-value** |
| --- | --- | --- | --- |
| **Vasomotor** | 0 (0 – 0) | 0 (0 – 0) | 0.901 |
| **Secretomotor** | 0 (0 – 4.3) | 2.1 (0 – 6.4) | 0.368 |
| **Gastrointestinal** | 4.9 (1.8 – 8.9) | 6.3 (3.6 – 8) | 0.403 |

| **B** | **DMF**  **(N = 23)**  **Median (IQR)** | **Untreated**  **(N = 97)**  **Median (IQR)** | **P-value** |
| --- | --- | --- | --- |
| **Vasomotor** | 0 (0 – 1) | 0 (0 – 0) | 0.777 |
| **Gastrointestinal** | 4.5 (0.9 – 7.1) | 6.3 (3.6 – 8) | 0.154 |

| **C** | **FTY**  **(N = 16)**  **Median (IQR)** | **Untreated**  **(N = 97)**  **Median (IQR)** | **P-value** |
| --- | --- | --- | --- |
| **Orthostatic** | 12 (0 – 18) | 8 (0 – 16) | 0.090 |

IQR = interquartile range
